# Supplementary figures and images for: Efficacy of JOINS Tablet for Lumbar Spinal Stenosis: Prospective, Randomized, Open-Label Clinical Trial
Source: Medicina (Kaunas). 2025 Oct 23;61(11):1900. doi: 10.3390/medicina61111900 (PMC12654109; doi:10.3390/medicina61111900)

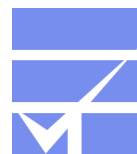

# CONSORT

TRANSPARENT REPORTING of TRIALS

## CONSORT 2010 Flow Diagram

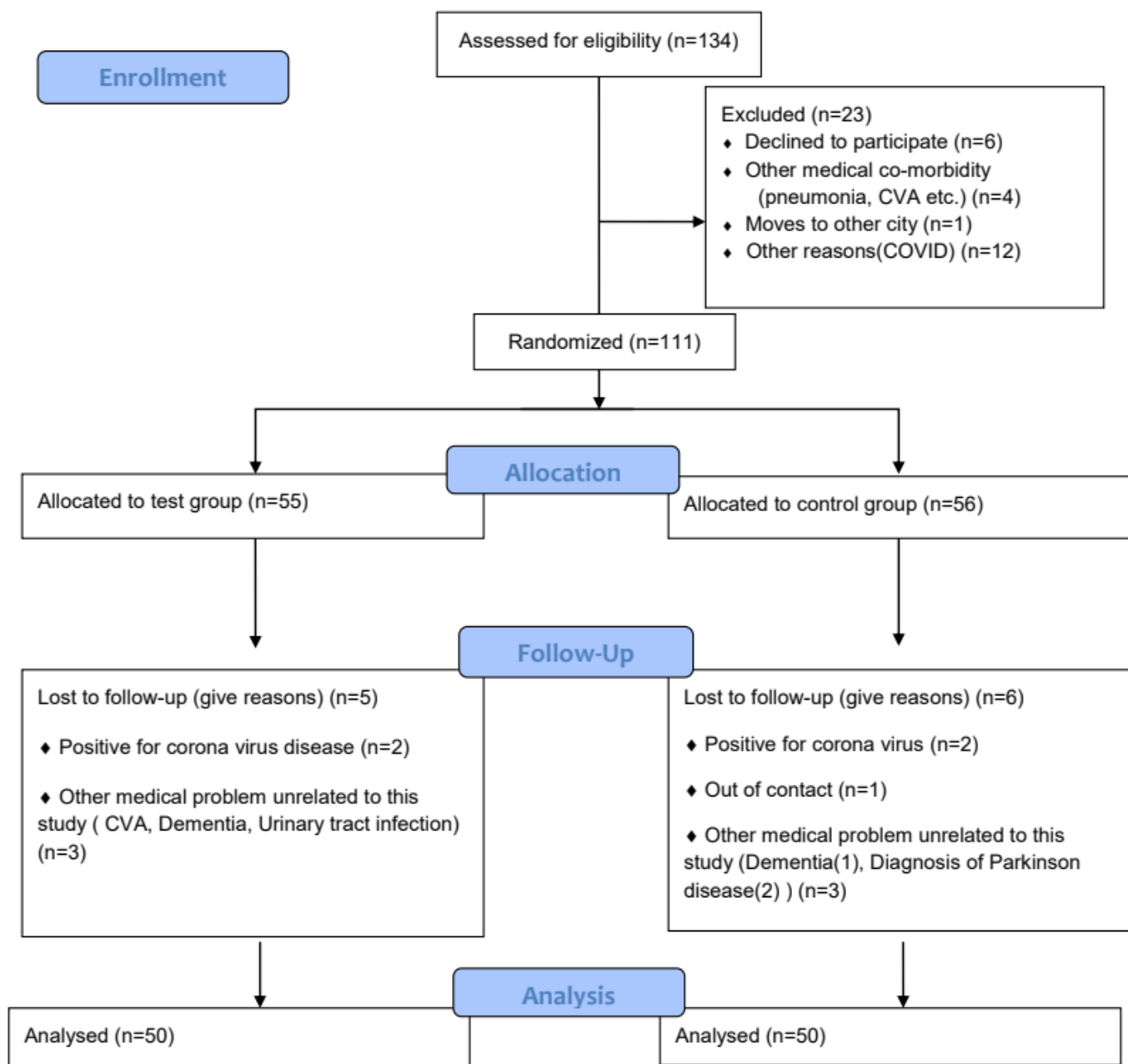

Supplement: Supplementary file 1 [file medicina-61-01900-s001.zip › Figure_S1.pdf]
